# Supplementary material for: Strong repulsive Lifshitz-van der Waals forces on suspended graphene
Source: Nat Commun. 2025 Aug 19;16:7726. doi: 10.1038/s41467-025-63074-1 (PMC12365322; doi:10.1038/s41467-025-63074-1)
Supplement: Supplementary file 1 — Supplementary Information [file 41467_2025_63074_MOESM1_ESM.pdf]

Supplementary Material for:
Strong Repulsive Lifshitz-van der Waals Forces on Suspended Graphene

Gianluca Vagli *[1], Tian Tian *[1,2], Franzisca Naef[1], Hiroaki Jinno †[1], Kemal Celebi[1], Elton J. G. Santos[3,4], and Chih-Jen Shih ‡[1]

[1]Institute for Chemical and Bioengineering, ETH Zürich, CH-8093 Zürich, Switzerland

[2]Department of Chemical and Materials Engineering, University of Alberta, Alberta, Canada

[3]Institute for Condensed Matter Physics and Complex Systems, School of Physics and Astronomy, The University of Edinburgh, EH9 3FD, UK.

[4]Higgs Centre for Theoretical Physics, The University of Edinburgh, EH9 3FD, United Kingdom

*These authors contributed equally to this work.

†Present adress: Institute of Space and Astronautical Science, JAXA, 3-1-1 Yoshinodai, Chuo-ku, Sagamihara-shi, Japan

‡Corresponding author. Email: chih-jen.shih@chem.ethz.ch

Suppl. Note 1 Theoretical Simulations

1.1 Modified Lifshitz theory for anisotropic media

The derivation of equation (2) is described as follows. The vdW interaction energy $\Phi_{\mathrm{AmB}}^{\mathrm{vdW}}$ corresponding to the total energy summed from all allowed EM modes[1] is given by:

$$\Phi_{\mathrm{AmB}}^{\mathrm{vdW}} = \frac{k_{\mathrm{B}}T}{2(2\pi)^2} \sum_{n=-\infty}^{\infty} \int_{r_n}^{\infty} \ln \mathcal{D}(i\xi_n, \mathbf{k}) d^2\mathbf{k} \tag{S1}$$

where $\mathbf{k} = (k_x, k_y)$ is the in-plane wavevector, and $\mathcal{D}(i\xi_n, \mathbf{k})$ is the dispersion relation for a given geometry. For generality, the dielectric tensor of material j has diagonal components $\varepsilon_{\mathrm{j}}^{xx}$, $\varepsilon_{\mathrm{j}}^{yy}$ and $\varepsilon_{\mathrm{j}}^{zz}$. By transforming $\mathbf{k} = (\kappa \cos\vartheta, \kappa \sin\vartheta)$, and $\mathrm{g_j} = \left[\frac{\varepsilon_{\mathrm{j}}^{xx}}{\varepsilon_{\mathrm{j}}^{zz}} \cos^2\vartheta + \frac{\varepsilon_{\mathrm{j}}^{yy}}{\varepsilon_{\mathrm{j}}^{zz}} \sin^2\vartheta\right]^{-1}$ where κ, ϑ are the corresponding polar coordinates of $\mathbf{k}$, the dispersion relation $\mathcal{D}$ of an anisotropic A/m/B layered system follows[2]:

$$\begin{aligned} \mathcal{D} &= 1 - \underbrace{\left[\frac{\hat{\varepsilon}_{\mathrm{A}} - \varepsilon_{\mathrm{m}}^{zz} g_m^{1/2}(\vartheta)}{\hat{\varepsilon}_{\mathrm{A}} + \varepsilon_{\mathrm{m}}^{zz} \mathrm{g_m}^{1/2}(\vartheta)}\right]}_{\Delta_{\mathrm{Am}}} \\ &\quad \times \underbrace{\left[\frac{\hat{\varepsilon}_{\mathrm{B}} - \varepsilon_{\mathrm{m}}^{zz} \mathrm{g_m}^{1/2}(\vartheta)}{\hat{\varepsilon}_{\mathrm{B}} + \varepsilon_{\mathrm{m}}^{zz} \mathrm{g_m}^{1/2}(\vartheta)}\right]}_{\Delta_{\mathrm{Bm}}} e^{-2\mathrm{g_m}^{1/2}(\vartheta)\kappa z} \\ &= 1 - \Delta_{\mathrm{Am}}(\vartheta)\Delta_{\mathrm{Bm}}(\vartheta) e^{-2\mathrm{g_m}^{1/2}(\vartheta)\kappa z} \end{aligned} \tag{S2}$$

By further introducing an auxiliary variable $x = 2\mathrm{g_m}^{1/2}(\theta)\kappa z$, it follows:

$$\Phi_{\mathrm{AmB}}^{\mathrm{vdW}} = \frac{k_{\mathrm{B}}T}{32\pi^2 z^2} \sum_{n=-\infty}^{\infty} \int_0^{2\pi} \mathrm{g_m}(i\xi_n, \vartheta) d\vartheta \int_{r_n}^{\infty} x dx \ln[1 - \Delta_{\mathrm{Am}}(i\xi_n, \vartheta)\Delta_{\mathrm{Bm}}(i\xi_n, \vartheta)e^{-x}] \tag{S3}$$

Accordingly, equation (2) is obtained for $\varepsilon_{\mathrm{m}}^{xx} = \varepsilon_{\mathrm{m}}^{yy}$, i.e. $\mathrm{g_m}$ is independent of ϑ, which is valid for most 2D materials where the 2D lattice is hexagonal or square. Moreover, in equation (2), $\hat{\varepsilon}_{\mathrm{A}}$ and $\hat{\varepsilon}_{\mathrm{B}}$ reduce to ε_{A} and ε_{B}, respectively, when A and B are isotropic bulk materials. Time-reversal symmetry $\varepsilon(i\xi) = \varepsilon(-i\xi)$ is used when magnetic response of the material is negligible, therefore equation (2) only needs to be evaluated for $\xi_n \geq 0$. Moreover, our numerical analysis suggests the integral of $\Phi_{\mathrm{AmB}}^{\mathrm{vdW}}$ is dominated by $x \leq 5$, or equivalently $\kappa \leq 2.5(\mathrm{g_m}d)^{-1}$. When d is in the order of 2 nm, and $\mathrm{g_m} = 2.5$, the majority of interaction comes from EM modes with $\kappa < 0.05$ Å^{-1}. In other words, evaluating equation (2) using the material dielectric functions at

the optical limit ($\mathbf{k} \rightarrow 0$) would preserve the accuracy of Φ_{AmB} calculation.

The vdW force per unit area $F^{\mathrm{vdW}}_{\mathrm{AmB}}$ is the derivative of the vdW potenial with respect to z given by:

$$\begin{aligned} \mathbf{F}^{\mathrm{vdW}}_{\mathrm{AmB}} &= -\nabla\Phi_{\mathrm{AmB}} = -\frac{\partial\Phi^{\mathrm{vdW}}_{\mathrm{AmB}}}{\partial z}\cdot\hat{\mathrm{e}}_z \\ &= \sum_{n=-\infty}^{\infty}\frac{k_{\mathrm{B}}Tg_{\mathrm{m}}(i\xi_n)}{16\pi z^3}\left\{\int_{r_n}^{\infty}x^2\frac{\Delta_{\mathrm{Am}}(i\xi_n)\Delta_{\mathrm{Bm}}(i\xi_n)e^{-x}}{1-\Delta_{\mathrm{Am}}(i\xi_n)\Delta_{\mathrm{Bm}}(i\xi_n)e^{-x}}dx\right\}\cdot\hat{\mathrm{e}}_z \end{aligned} \quad (S4)$$

which leads us to equation (3) for the vdW force per unit area $F^{\mathrm{vdW}}_{\mathrm{AmB}} = |\mathbf{F}^{\mathrm{vdW}}_{\mathrm{AmB}}|$.

Complementary to Fig. 1b, Fig. 1 compares the dielectric responses of other materials studied here. Notably, at the order of $d = 1$ nm, $\hat{\varepsilon}_{\mathrm{m}}$ of graphene is comparable to that of bromobenzene (BB), a high-refractive-index liquid commonly used in experiments demonstrating Casimir repulsion[3, 4]. In this respect, 2D material appears to be a promising candidate for realizing repulsive vdW interactions. However, unlike bulk liquid, $\hat{\varepsilon}_{\mathrm{m}}$ of a 2D material strongly depends on the separation d, making the repulsion more pronounced at short distances (the vdW regime).

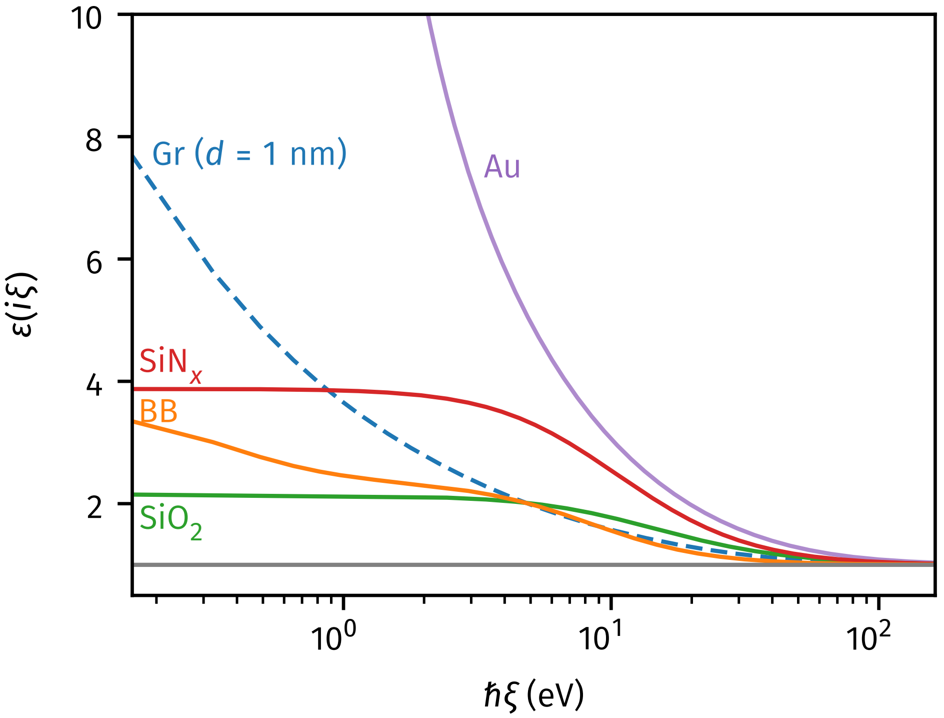

Suppl. Fig. 1. **Comparison of $\hat{\varepsilon}(i\xi)$ responses for materials considered in this study**. The effective dielectric function of graphene at $d = 1$ nm is higher than that of the widely-used high-refractive-index liquid bromobenzene (BB).

We further compared the force response $F^{\mathrm{vdW}}_{\mathrm{AmB}}$ for A/m/B = Vac/Gr/Au, Vac/Gr/$\mathrm{SiN_x}$ and

SiO_2/Bromobenzene(BB)/Au, in which the latter quantitatively demonstrates Lifshitz-Casimir repulsion at large separations [4]. We can cleary see that the three systems are very representative, generating repulsion in the vdW and Casimir interaction regimes, respectively. Figure 2 shows that for 30 nm, all three interactions are repulsive with similar strength, with the liquid immersion system being slightly larger for z larger than 50 nm, resulting from a reduction of graphene's dielectric response. Nevertheless, as pointed out by Boström et al [5], in SiO_2/BB/Au system, the receding retardation effect turns the Casimir repulsion to vdW attraction for $z < 20$nm, exhibiting a maximum repulsive force of $15 \, \frac{\mathrm{N}}{\mathrm{m}^2}$ at approximately 25 nm.

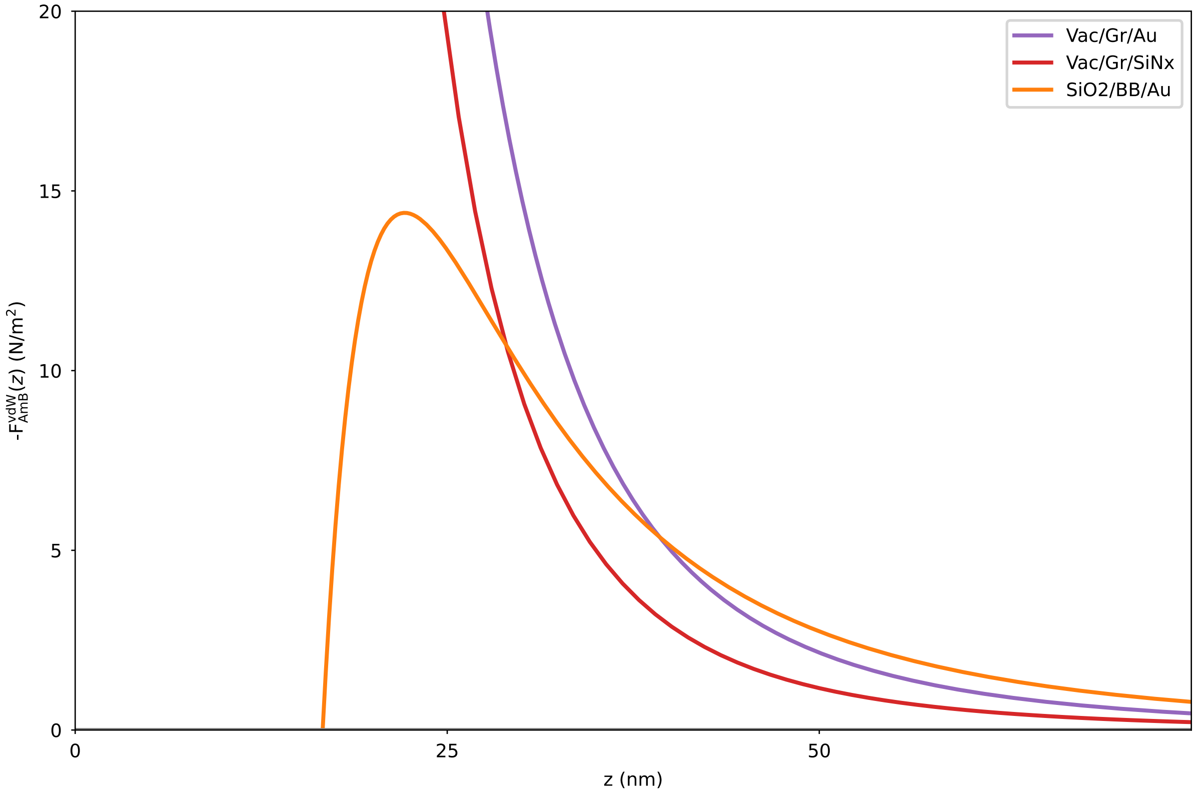

Suppl. Fig. 2. **Comparison of $F^{\mathrm{vdW}}_{\mathrm{AmB}}$ for A/m/B = Vac/Gr/Au, Vac/Gr/SiN_x and SiO_2/Bromobenzene(BB)/Au.** For $z > 30$ nm, all three interactions are repulsive with similar strength, with the liquid immersion system being slightly larger for $z > 50$ nm. As pointed out by Boström et al [5], in SiO_2/BB/Au system, the receding retardation effect turns the Casimir repulsion to vdW attraction for small separations z.

Suppl. Note 2 Force-distance AFM measurements

2.1 AFM tip size

Figure 3 shows magnified SEM images of the AFM probes considered in this study. The hemispherical shape of the tip is highlighted with a red dashed line and the radius R was estimated to be approximately (Fig. 3a) 33 nm and (Fig. 3b) 13 nm for the gold-coated tips with respective calibrated deflection sensitivities of 69.679 $\frac{\text{nm}}{\text{V}}$, 86.484 $\frac{\text{nm}}{\text{V}}$ and spring constants of 0.16136 $\frac{\text{N}}{\text{m}}$, 0.14710 $\frac{\text{N}}{\text{m}}$ and (Fig. 3c) 20 nm for the uncoated tip with a calibrated deflection sensitivity of 75.567 $\frac{\text{nm}}{\text{V}}$ and a spring constant of 0.11670 $\frac{\text{N}}{\text{m}}$.

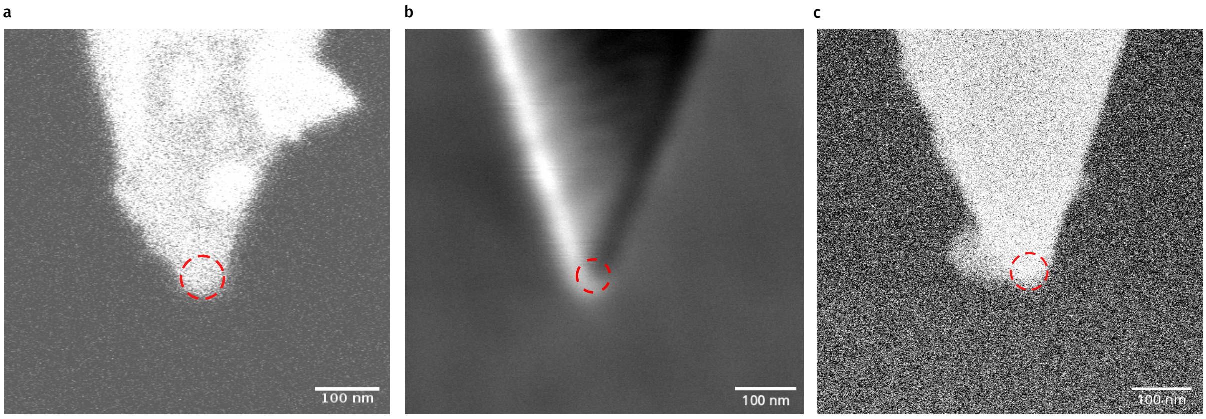

Suppl. Fig. 3. **Magnified SEM images of the AFM probes considered in this study.** The hemispherical shape of the tip is highlighted with a red dashed line and the radius R was estimated to be approximately **a** 33 nm and **b** 13 nm for the gold-coated tips with respective calibrated deflection sensitivities of 69.679 $\frac{\text{nm}}{\text{V}}$, 86.484 $\frac{\text{nm}}{\text{V}}$ and spring constants of 0.16136 $\frac{\text{N}}{\text{m}}$, 0.14710 $\frac{\text{N}}{\text{m}}$ and **c** 20 nm for the uncoated tip with a calibrated deflection sensitivity of 75.567 $\frac{\text{nm}}{\text{V}}$ and a spring constant of 0.11670 $\frac{\text{N}}{\text{m}}$.

2.2 AFM background noise subtraction

The raw measurement data was processed by subtracting the background noise, which was determined by fitting the force-distance measurement data with the following function:

$$f_{\text{Bkg}}(d) = p_0 + p_1 d + A \sin(\omega d + \theta) \tag{S5}$$

within the displacement range d between 150 nm to 450 nm, which is far before the tip establishes contact or after it retracts from graphene. The function f_{Bkg} contains two distinct parts of

correction. The linear function $p_0 + p_1 d$, could correct the noise resulting from any alignment issue in the measurement between tip and photodiode. The periodic function $A \sin(\omega d + \theta)$ is to correct the optical interference noise caused by stray light reflected from the supporting material, which is a well-known phenomena taking place during force-displacement measurement of reflective surfaces[6], [7]. This noise subtraction method is applied on both freestanding

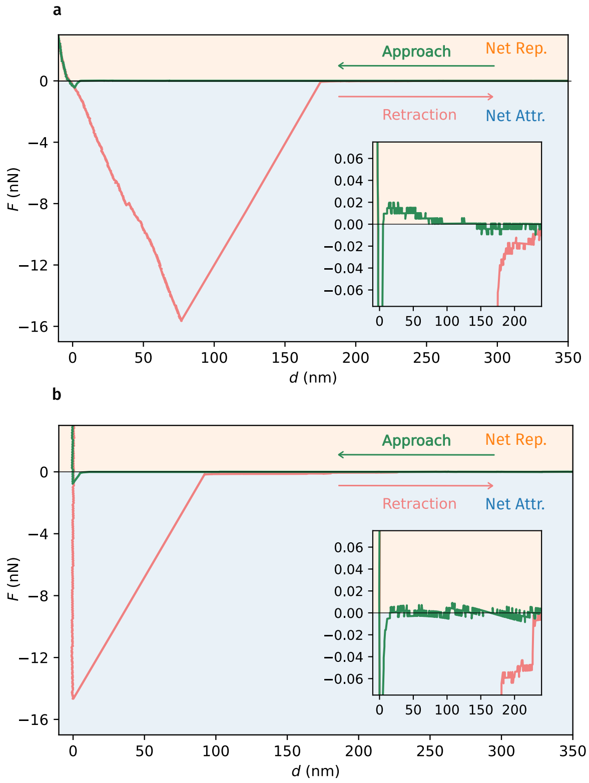

Suppl. Fig. 4. **Representative force-displacement responses after noise substraction of the Au-coated AFM tip.** **a** and **b** correspond to one force-displacement hysteresis measurement on freestanding and SiN_x supported graphene, respectively. The insets magnify the force responses within the contact region. Each measurement contains the approach (green) and retraction (red) responses.

and SiN_x supported graphene for both AFM tips, which yielded consistent results in terms of a stable baseline for further quantification, regardless of the substrate choice and whether the tip approach/retraction measurements. An example of processed force-displacement hysteresis measurement is shown in Fig. 4, where Fig. 4a and 4b correspond to measurements on freestanding and SiN_x supported graphene, respectively. The distance $d = 0$ nm was set to be the point for which the force is minimal, while establishing contact. The noise-subtracted force responses exhibit well-defined zero baseline, with all essential information retained within the contact region, such as repulsive and attractive interaction, as well as the mechanical responses on freestanding and supported graphene upon retraction [8].

Examples of two datasets of 16 individual force-displacement measurements without optical interference correction are shown in Figure 5. Both sets belong to the two measurements using a gold-coated AFM tip of 33 nm (Fig. 5a) and 13 nm (Fig. 5b) radius respectively, used to generate the 2D histograms for Figure 2a in the manuscript and Figure 7a. Noise caused by the optical interference within the flat areas of graphene is smaller than the repulsive feedback. This is due to the fact that they are located within the center of the pore, in which almost no light is back reflected.

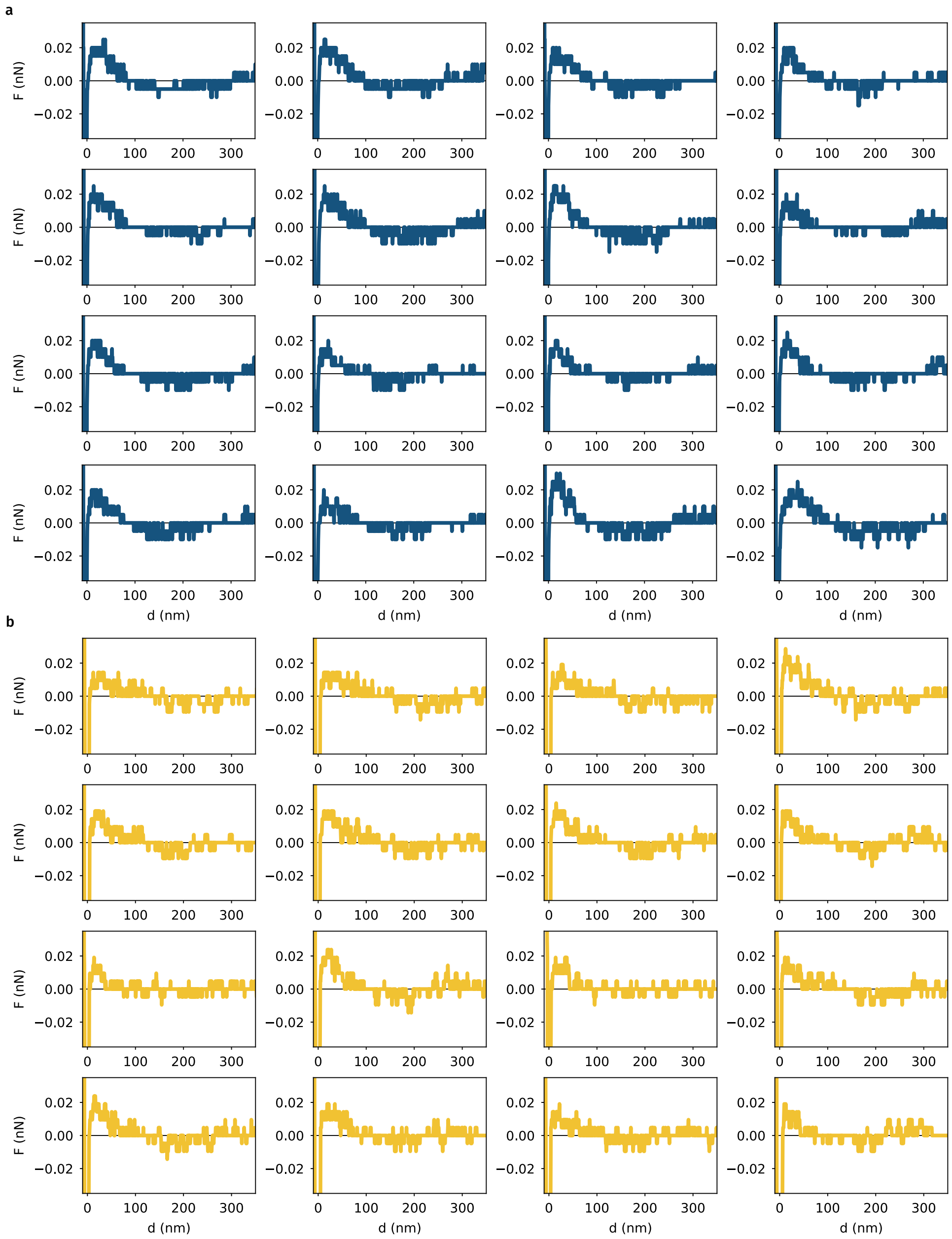

Suppl. Fig. 5. **16 individual force-displacement measurements without optical interference correction.** Both sets belong to the measurements used for the 2D histogram for **a** Figure 2a in the manuscript and **b** Figure 7a.

2.3 AFM topography and surface force analysis for corrugated domains

We evaluated the smaller repulsive region presented in Figure 1c, by plotting its cross sectional data similar to the ones presented in Figure 1f and 1g. The results are shown in Figure 6a. The smaller repulsive domain seems to be composed of convex graphene patches with sufficiently low surface gradient. The transition domains between the patches tend to either yield a weakened repulsion if none at all. This seem to further strengthen the argument of a certain maximum surface gradient threshold for which repulsion becomes detectable. If one analyses the area between the repulsive domains, presented in Figure 6b one can clearly see that it does not have any flat domain and thus does not exert any detectable repulsion.

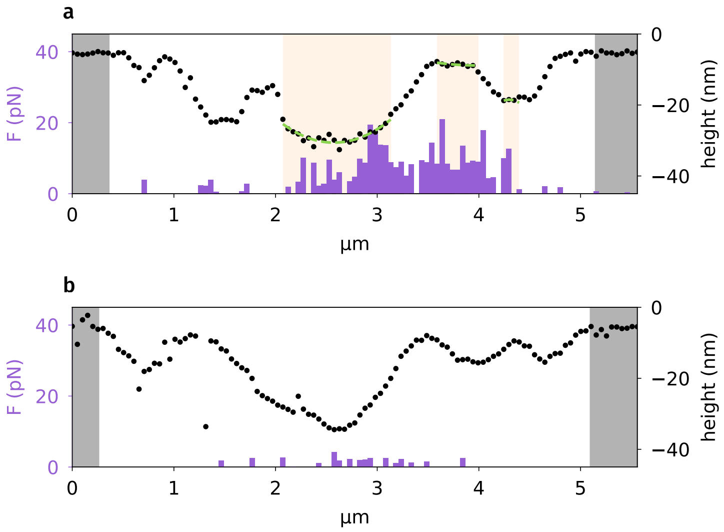

Suppl. Fig. 6. **Analysis of cross sectional datasets with respect to surface force data of corrugated domains from Figure 1b in the manuscript.a** Represents the cross sectional dataset of the smaller repulsive area found in 1c, while **b** represents the the cross sectional dataset between both small and large repulsive area. One can see in **a** that ripple shaped surface corrugations sometimes form convex surface areas with sufficiently low surface gradient,($\| \max\left(\frac{df_P}{dx}\right) \| \leq 0.01$ highlighted with the light orange shading) allowing the detection of repulsive forces, while an corrugated area without any flat domains like in **b** does not really exert any detectable repulsive feedback. The gray areas correspond to the boundaries of the SiN_x pore.

2.4 Lifshitz-vdW force-displacement response and analysis of smaller gold-coated AFM tip

Figure 7 shows the result of the force-displacement measurements on the gold-coated SiN_x using a gold-coated AFM tip with a radius of approximately 13 nm, as shown in Section 3. An

evaluation of 144 force-displacement curves of on flat suspended graphene within the white dashed circle using a 2D histogram, shown in Figure 7a yielded a repulsive force of 7.5 ± 5.7 pN was measured at a average separation of approximately 7.5 nm. Further analysis using the effective media approach, as presented in Section yielded an estimated effective radius R_{eff} and area of interaction A_{max} of 18nm and 4254 nm^2. The resulting maximum repulsive force is in proper scaling behavior to the larger repulsive force of 11.8 ± 4.6 pN measured using the gold-coated AFM tip with a radius of 33 nm. Furthermore the force is still significantly larger compared to the maximum repulsive force of 5.7 ± 4.0 pN measured using the bare SiN_x tip, which was observed at closer separations of 6.6 nm.

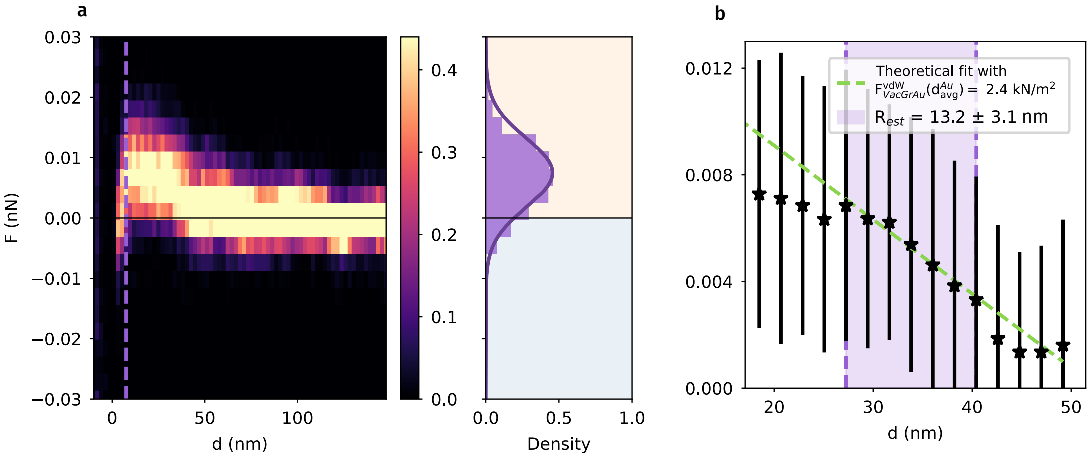

Suppl. Fig. 7. **Lifshitz-vdW force-displacement response of the gold-coated AFM tip with 13nm radius.a** 2D histogram of 144 force-displacement measurements taken from the center of the second sample. A repulsive force of 7.5 ± 5.7 pN was measured at a average separation of approximately 7.5 nm. The dashed line represents the last notable measured repulsive force before experiencing attraction. **b** If fitted with the same approach as in the main text for Figure 4 using the standard deviations of the histograms calculated from 144 measurements as error bars, this results in an estimated effective radius R_{eff} and area of interaction A_{max} of 18 nm and 4254 nm^2 respectively.

2.5 Force sensitivity and statistical significance of AFM data

The minimum detectable voltage of the photodiode detector is 0.375 mV. To obtain the respective force sensitivities for each cantilever, one needs to multiply the minimum detectable voltage of the photodiode detector by the AFM cantilever properties from Section 2.1. The resulting force sensitivities are 4.216 pN and 4.771 pN for the measurements using gold-coated tips of 30 nm and 13 nm radius, respectively, and 3.307 pN for the uncoated SiN_x tip. All prior information required for the calculations can be found listed in Table 1.

To prove the statistical significance of each measurement, a Welch t-test was conducted to

Measurement (Tip Radius)	Defl. Sensitivity [nm/V]	Spring Constant [N/m]	Resolution [pN] at 0.375 mV
Au-coated (30 nm)	69.679	0.16136	4.216
Au-coated (13 nm)	86.484	0.14710	4.771
Uncoated (20 nm)	75.567	0.11670	3.307

Suppl. Table. 1. **The estimation of force sensitivity for each AFM tip considered in this work.**

check whether both the signal and the noise floor level share the same mean as the null hypothesis. The significant amount of noise floor measurements allows one to distinguish it from the signal, enabling such a test in the first place. The choice to use Welch's t-test instead of Student's t-test was motivated by the fact that the variances of both signal and noise are not necessarily equal. If applied to the raw data without any periodic background subtraction of 2.2 in the SI, one gets p-values of 0.102 and 0.119 for both measurements using gold-coated tips with a radius of 30 nm and a radius of 13 nm, respectively. The corresponding histograms for this analysis are shown in Figure 8. Any evaluation before background subtraction for the bare SiN_x tip is not possible as the periodic background noise dominates the raw data, due to the material properties and the choice of the cantilever. Although not reaching the common statistical significance threshold $\alpha = 0.05$, the raw data in itself already show statistically distinct signs of being different from the noise floor.

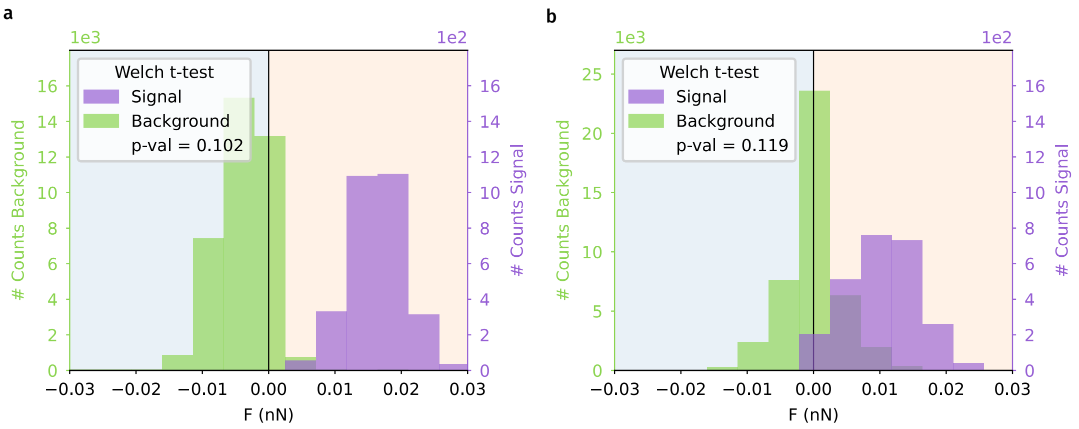

Suppl. Fig. 8. **Welch's t-test for signal and noise floors in the raw dataset.** Histograms for the measured repulsive (purple) and background (green) forces for the gold-coated tips of radii of 30 nm **a** and 13 nm **b** considered in this study. The extracted p-values are 0.102 and 0.119, respectively.

Once the period background noise is subtracted, p-values of 0.047 and 0.049 are obtained for both measurements using gold-coated tips of 30 nm and 13 nm radius, respectively, and 0.044 for the measurements using the bare SiN_x tip (Figure 9). All of these values are below the statistical significance threshold $\alpha = 0.05$, which allows the rejection of the null hypothesis that the signal and the noise floor share the same mean value, thus emphasizing the statistical significance of the measured signal.

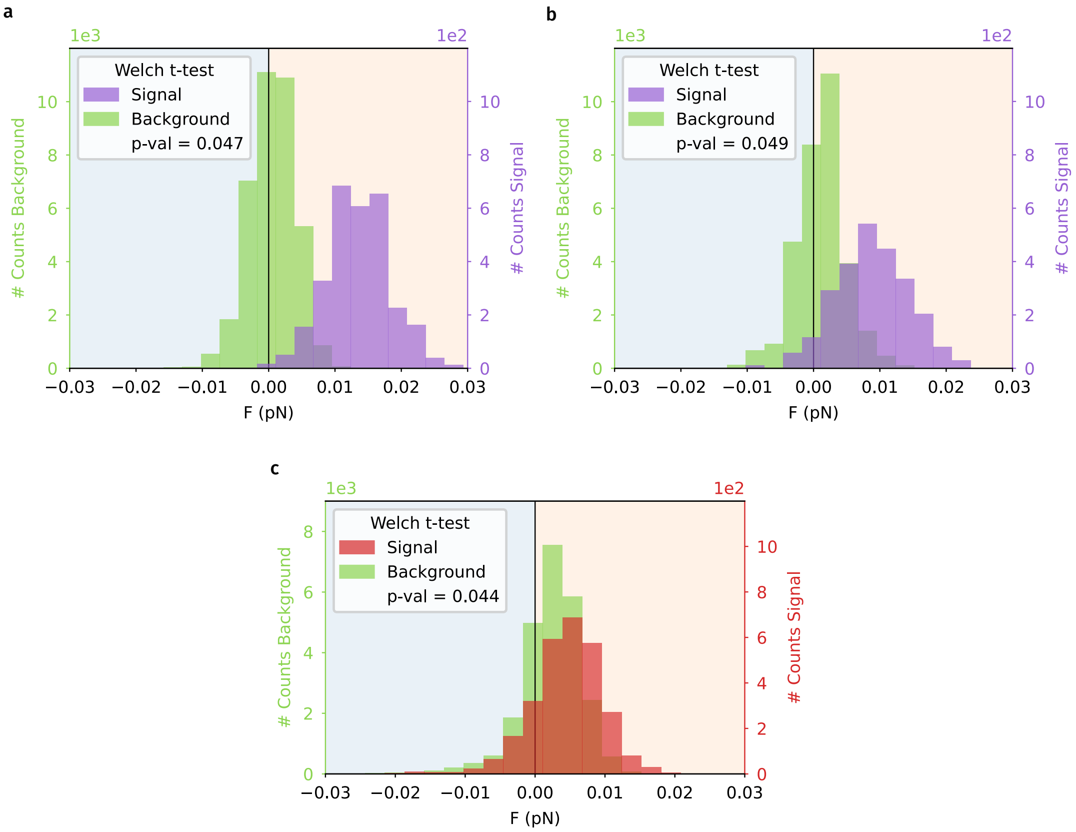

Suppl. Fig. 9. **Welch's t-test for signal and noise floors in the dataset after sinusoidal background subtraction.** Histograms for the measured repulsive (purple) and background (green) forces after sinusoidal background subtraction for the gold-coated tips of radii of 30 nm **a** and 13 nm **b**, as well as the bare SiNx tip **c** The extracted p-values are all below 0.05, consolidating the statistical significance of our measured repulsive forces.

Suppl. Note 3 Epitaxial experiments

3.1 Optical characterization of suspended graphene

Figure 10 shows an overlay of two optical images of the graphene sample before and after transfer onto a SiN_x holey membrane, to visualize the areas of varying thickness accurately.

A relative contrast enhancement of 4.8%, 16.3%, 25.3% and 35.6% was measured with respect to the background before transfer, indicating the presence of contrast enhancements of mono, bi-, tri-, and 4-layers that correspond to [9]. Two of the four areas of varying thickness yielded fully suspended areas. Those are highlighted in Figure 10a with blue and black dashed lines for monolayer and 4-layer graphene, respectively. The matching Raman spectroscopy measurements of these areas are shown in 10b and 10c, which confirm their respective thicknesses.

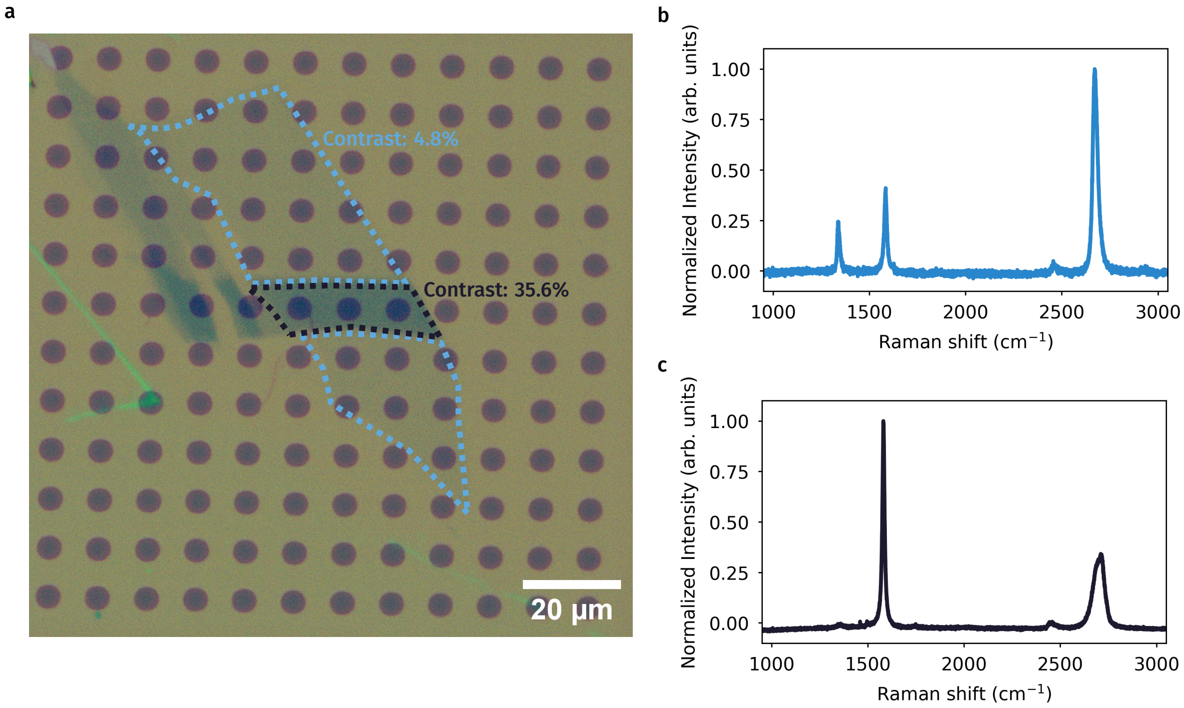

Suppl. Fig. 10. **The suspended graphene sample for the epitaxial nucleation experiment discussed in Fig. 5 in the main text.** **a** Overlay of the optical microscope images before and after transfer for the visualization of the areas of various graphene thicknesses. The values of relative contrast enhancement of 4.8%, 16.3%, 25.3%, and 35.6% were measured, allowing us to identify the mono-, bi-, tri-, and 4-layer graphene areas. Two large areas of suspended mono-and 4-layer graphene, corresponding to the enclosed blue and black dashed lines, were identified for statistical analysis shown in Fig. 5. **b, c** Representative Raman spectra for mono- **b** and 4-layer **c** graphene.

Suppl. Note 4 Additional Discussions

This section contains further discussions that are not directly related to the results in the main text but may help understanding or extending our current work.

4.1 Influence of bulk material bandgap

In principle, the vdW repulsion may also be observed on freestanding graphene even if the bulk material is semiconductor or insulator, as long as the inequality (1) in main text holds. Here we theoretically explore the influence of the bandgap of bulk material on the interfacial vdW forces in a Vac/Gr/B system, where B is the bulk material with varied bandgap. Fig 11a compared the frequency-dependent dielectric functions for typical metal (Au), semiconductors (GaAs, GaN) and insulator (SiO_2) as compared with graphene at $d = 1$ nm. As a general trend, the dielectric response decreases for materials with larger bandgap. As a result, the interaction spectra for Vac/Gr/B systems gradually shift to attractive at lower frequencies, when the bandgap of B increases (Fig. 11b and 11c). The results indicate that on freestanding graphene interface, it may be easiest to observe the vdW repulsion if material B is a metal, compared to semiconductors or insulators.

4.2 Influence of 2D material layer number

An intuitive way to control the degree of interfacial forces on 2D materials is to control their layer numbers, which can also be captured by our theoretical framework. We model the many-body vdW interactions by extending the analysis with the layered planar system description from Parsegian's work [2]. For a system of Vac/NL-Gr/Au where NL-Gr represents N-layer graphene stacks, for small N, we assume that the polarizability of NL-Gr α^{p}_{NL} linearly scales with N [10], such that

$$\alpha^{p}_{\mathrm{NL}} = N\alpha^{p}_{\mathrm{2D}} \tag{S6}$$

where p is either in- or out-of-plane components and α_{2D} is the two-dimensional electronic polarizability of monolayer graphene.

Using this model, we calculated the distance-dependent total vdW interaction energy Φ_{tot} for the Vac/NL-Gr/Au system with varied finite layer numbers, as shown in Fig. 12. As expected, with increasing layer number of graphene, the repulsive interaction decreases. We propose that such change can be probed by AFM on freestanding graphene with controlled layer numbers, which can be prepared using mechanical exfoliation techniques.

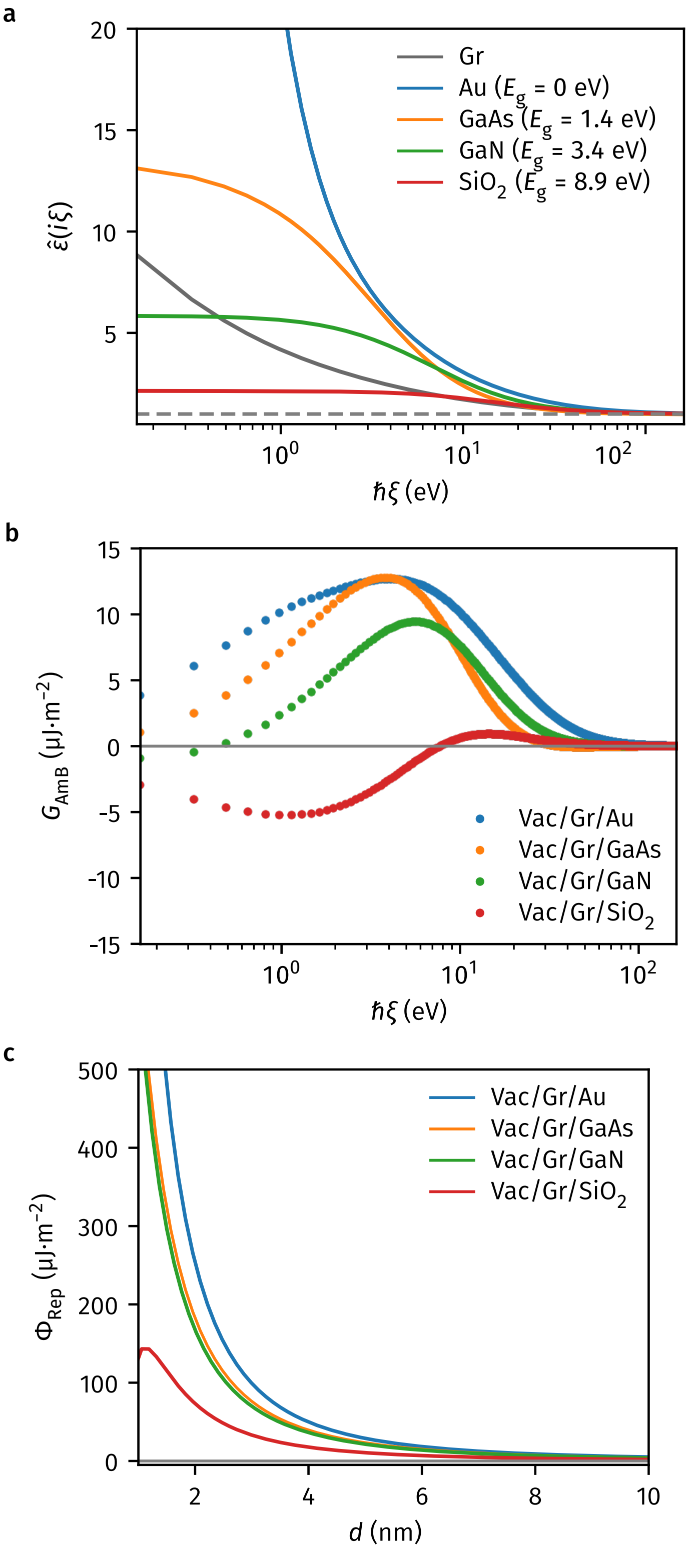

Suppl. Fig. 11. **Influence of bulk material bandgap on the interfacial vdW repulsion a**. Dielectric functions for Au, GaAs, GaN and SiO_2 compared with graphene at d = 1 nm. **b**. Interaction spectra of Vac/Gr/B systems when B=Au, GaAs, GaN and SiO2, respectively. **c**. Distance-dependent vdW repulsion energy of corresponding system in **b**.

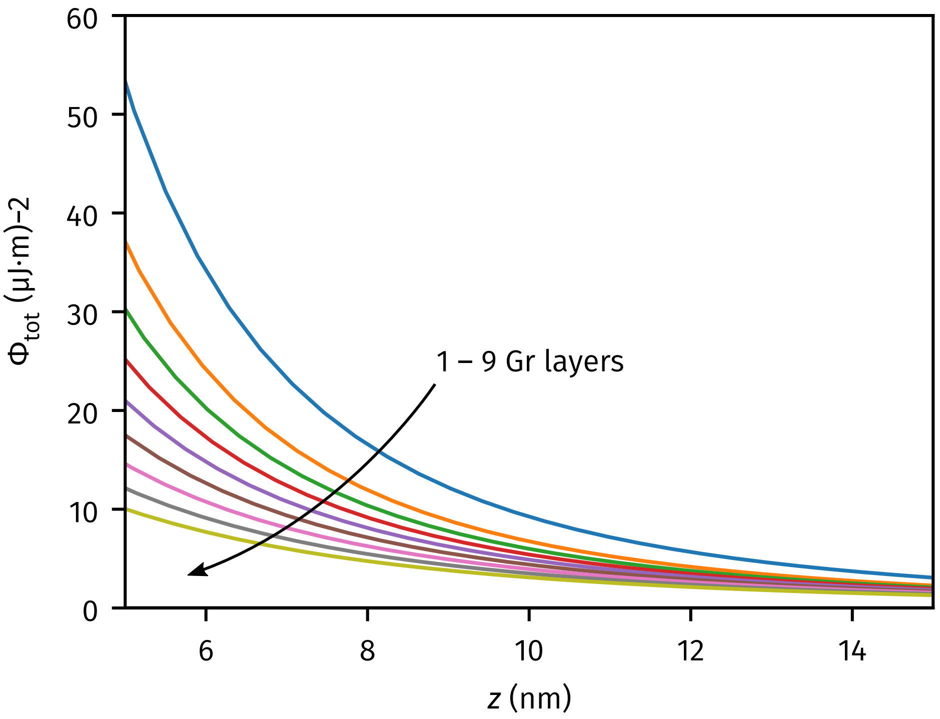

Suppl. Fig. 12. **Total vdW interaction energy for Vac/NL-Gr/Au system with various graphene layer numbers.** With increasing graphene layer thickness, the repulsive response diminishes.

4.3 Choice of 2D material

Another degree of freedom for controlling the interfacial forces is the choice of 2D material; for example, replacing graphene with MoS_2 or hBN. Eq. (S3) shows that Φ^{vdW}_{AmB} depends on:

1. **Dielectric mismatch**: $\Delta_{Am}\Delta_{Bm}$
2. **Dielectric anisotropy of *m***: g_m

For isotropic medium m, $g_m = 1$. The maximal vdW repulsion at a given frequency ξ occurs:

$$\frac{\partial \Delta_{Am}(\xi)\Delta_{Bm}(\xi)}{\partial \hat{\varepsilon}_m(\xi)} = 0 \tag{S7}$$

by definition of Δ_{Am} and Δ_{Bm} this is equivalent to $\hat{\varepsilon}_m(\xi) = \sqrt{\varepsilon_A(\xi)\varepsilon_B(\xi)}$. However, using a 2D material as medium, we have $g_m < 1$, and g_m becomes smaller when the bandgap of 2D material decreases[10]. Therefore the situation is more complex than with homogeneous medium. Our theoretical analysis in Figure 13 shows changes in frequency dependent energy contributions if effects of 2D anisotropy are considered. To show this we selected both MoS_2 and hBN as they are commonly found 2D materials within the vdW heterostructure research

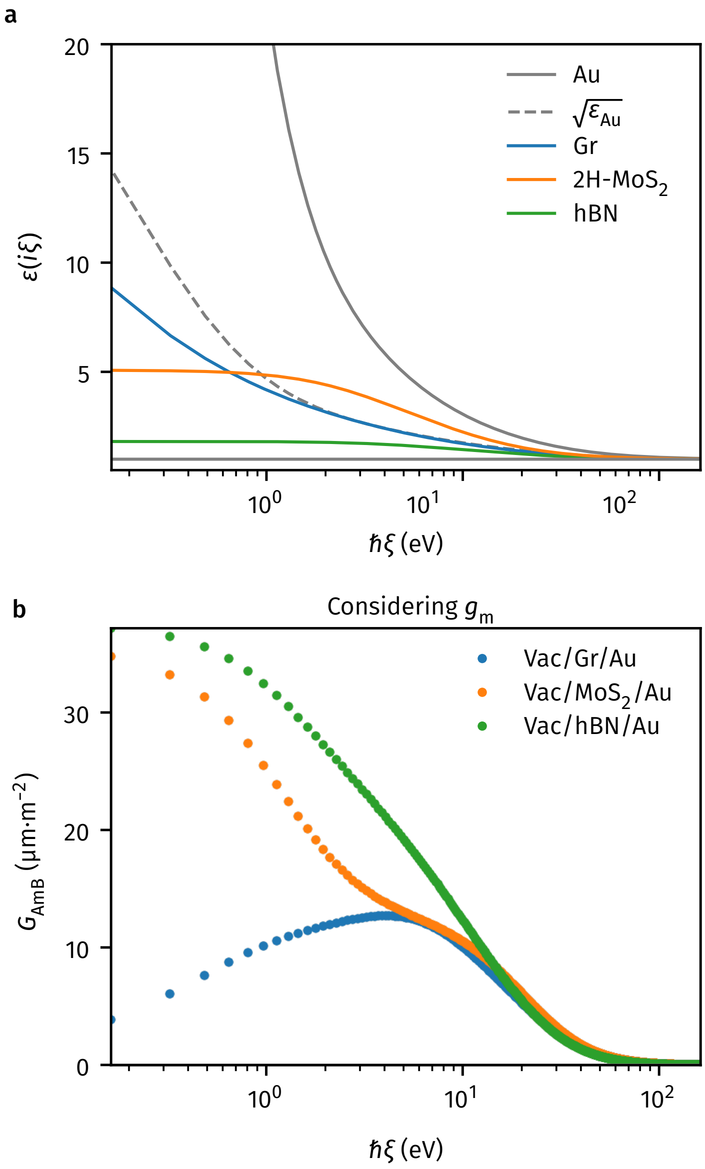

Suppl. Fig. 13. **Influence of choice of 2D material.** **a**. Frequency-dependent dielectric responses for graphene, MoS_2, hBN at d = 1 nm. **b** Calculated interaction spectra taking into account g_m, revealing that the Vac/hBN/Au system could yield stronger vdW repulsion.

and are good representative for graphene-like materials with semiconducting and insulating properties respectively, while having a non-planar geometry. Figure 14 show the resulting repulsive interaction calculated for previously mentioned materials.

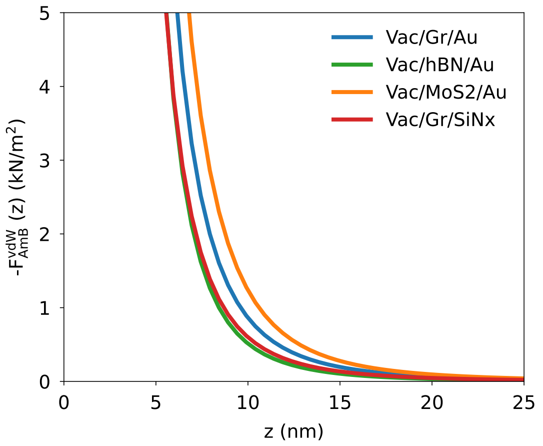

Suppl. Fig. 14. **Comparison of the calculated interaction force for different suspended 2D monolayers.** One can observe that the suspended MoS_2 yields up to a 43% stronger repulsive force than graphene within the measurement-relevant regime (6 – 12 nm).

While we expected hBN to yield a stronger repulsive response, given the higher low frequency contributions found in Figure 13 it turned out that MoS_2 is stronger within our usually observed measurement range of 6-12nm. In fact within this range hBN seems to behave similar to a Vac/Gr/SiN_x system, which tells us that the anisotropicity factor g_m is only affecting the interaction at separation z of at minimum 1 nm. MoS_2 shows indeed up tp 43% stronger repulsion at same separations than graphene, which is a separation correction similar to the experimental results of a Vac/Gr/SiN_x system if compared to a Vac/Gr/Au, with equal trend.

Supplementary References

1. Li, J.-L. **andothers**. Use of dielectric functions in the theory of dispersion forces. *Phys. Rev. B* **71,** 235412 (2005).
2. Parsegian, V. A. *Van der Waals Forces: A Handbook for Biologists, Chemists, Engineers, and Physicists* Cambridge Core. (2017).
3. Meurk, A., Luckham, P. F. & Bergström, L. Direct Measurement of Repulsive and Attractive van der Waals Forces between Inorganic Materials. *Langmuir* **13,** 3896–3899 (1997).
4. Munday, J. N., Capasso, F. & Parsegian, V. A. Measured long-range repulsive Casimir–Lifshitz forces. *Nature* **457,** 170–173 (2009).
5. Boström, M., Sernelius, B. E., Brevik, I. & Ninham, B. W. Retardation turns the van der Waals attraction into a Casimir repulsion as close as 3 nm. *Phys. Rev. A* **85,** 010701 (2012).
6. Weisenhorn, A. L., Maivald, P., Butt, H.-J. & Hansma, P. K. Measuring adhesion, attraction, and repulsion between surfaces in liquids with an atomic-force microscope. *Phys. Rev. B* **45,** 11226–11232 (19 1992).
7. Kassies, R., van der Werf, K. O., Bennink, M. L. & Otto, C. Removing interference and optical feedback artifacts in atomic force microscopy measurements by application of high frequency laser current modulation. *Review of Scientific Instruments* **75,** 689–693 (2004).
8. Lee, C., Wei, X., Kysar, J. W. & Hone, J. Measurement of the Elastic Properties and Intrinsic Strength of Monolayer Graphene. *Science* **321,** 385–388 (2008).
9. Thomsen, J. D. **andothers**. Suspended Graphene Membranes to Control Au Nucleation and Growth. *ACS Nano* **16.** PMID: 35849654, 10364–10371. eprint: `https://doi.org/10.1021/acsnano.2c00405` (2022).
10. Tian, T. **andothers**. Electronic Polarizability as the Fundamental Variable in the Dielectric Properties of Two-Dimensional Materials. *Nano Lett.* **20,** 841–851 (2019).
